# Acceptability and Feasibility of a Mindfulness Intervention Delivered via Videoconferencing for People With Parkinson’s

**DOI:** 10.1177/0891988720988901

**Published:** 2021-01-28

**Authors:** Angeliki Bogosian, Catherine S. Hurt, John V. Hindle, Lance M. McCracken, Debora A. Vasconcelos e Sa, Sandra Axell, Katy Tapper, Jemima Stevens, P. Shashi Hirani, Marya Salhab, Wenrong Ye, Patricia Cubi-Molla

**Affiliations:** 1 Division of Health Services Research and Management, 4895 City, University of London, London, United Kingdom; 2 School of Psychology, Bangor University, Bangor, United Kingdom; 3 Institute of Psychiatry, Psychology & Neuroscience, King’s College London, London, United Kingdom; 4 School of Psychology, 4895 City, University of London, London, United Kingdom; 5 Amaris Technology Consulting Co., Ltd, Huang Pu District Shanghai; 6 60882 Office of Health Economics, London, United Kingdom

**Keywords:** Parkinson’s, mindfulness, anxiety, depression, EQ-5D

## Abstract

Mindfulness-based group therapy is a rapidly growing psychological approach that can potentially help people adjust to chronic illness and manage unpleasant symptoms. Emerging evidence suggests that mindfulness-based interventions may benefit people with Parkinson’s. The objective of the paper is to examine the appropriateness, feasibility, and potential cost-effectiveness of an online mindfulness intervention, designed to reduce anxiety and depression for people with Parkinson’s. We conducted a feasibility randomized control trial and qualitative interviews. Anxiety, depression, pain, insomnia, fatigue, impact on daily activities and health-related quality of life were measured at baseline, 4, 8, and 20 weeks. Semi-structured interviews were conducted at the end of the intervention. Participants were randomized to the Skype delivered mindfulness group (n = 30) or wait-list (n = 30). Participants in the mindfulness group were also given a mindfulness manual and a CD with mindfulness meditations. The intervention did not show any significant effects in the primary or secondary outcome measures. However, there was a significant increase in the quality of life measure. The incremental cost-effectiveness ratio was estimated to be £27,107 per Quality-Adjusted Life Year gained. Also, the qualitative study showed that mindfulness is a suitable and acceptable intervention. It appears feasible to run a trial delivering mindfulness through Skype, and people with Parkinson’s found the sessions acceptable and helpful.

## Introduction

Anxiety and depression are prevalent in people with Parkinson’s (PwP). Depending on the criteria used, depression affects up to 50% of PwP,^1^ and up to 31% report some level of anxiety.^2^ Even when PwP do not experience significant psychological disorders, they may still struggle to adjust to the social, emotional and personal changes brought on by the condition.

Recent reviews and meta-analyses have shown that cognitive behavioural therapy (CBT) interventions are promising treatment approaches when addressing psychological distress in Parkinson’s.^3-8^ Due to mobility limitations, travel burden and cost, psychological treatment can be inaccessible in Parkinson’s, and the conditions have definitely been intensified by the implementation of lockdown during the COVID-19 pandemic.^9^ Therefore, there has been a recent shift toward delivering care remotely.^10^ Preliminary evidence shows that remotely delivered CBT interventions can improve psychological wellbeing for people affected by Parkinson’s.^11-13^

Further, mindfulness-based group therapy, typically an 8-week course of weekly meetings and daily mindfulness meditation practice, is a rapidly growing psychological approach that can potentially help people adjust to chronic illness and manage unpleasant symptoms.^14^ Mindfulness focuses on finding a new way to relate to thoughts and experiences and to accept them as passing events that do not necessarily represent a state of reality.^15^ Mindfulness is based on the philosophy that human suffering develops in part by efforts to struggle with and avoid our own psychological and emotional pain. Changing one’s relationship to thoughts appears to be the most active component of mindfulness-based interventions (MBIs).^15^

Emerging evidence suggests that MBIs may benefit the PwP. Four recent small randomized control trials (n = 29, 30, 14, and 36) of MBI showed significant decreases in motor symptoms^16-18^ symptoms of depression,^16,19,20^ and symptoms of anxiety^16^ for PwP and showed an increase in gray matter density in the hippocampus and amygdala.^17^ Importantly, a qualitative study evaluating the acceptability and feasibility of this approach in a group of PwP has found that this form of intervention is well accepted and described as both challenging and life-enhancing.^21^ In addition, MBI have frequently been found to provide good value for money, being cost-saving treatments while improving health-related quality of life (HRQoL) for distressed patients.^14,22^

We delivered a MBI via Skype, an online application that enables video conferences with 2 or more people. There is evidence that distant delivered MBIs can be effective in people with chronic medical conditions.^10,14,23-25^ The overall objective of this study was to assess the potential feasibility and acceptability of an MBI for PwP delivered via Skype.

The specific aims of this trial were to:

1. Examine recruitment, retention, and attendance to the mindfulness sessions to establish the feasibility of the current protocol for future larger-scale efficacy trials.
2. Evaluate the potential efficacy of the MBI regarding improvements in anxiety, depression, pain, insomnia, fatigue, and daily activities.
3. Evaluate the potential effectiveness of the distant delivered MBI regarding improvements in HRQoL measures and provide a rough estimate of the cost-effectiveness of the intervention.
4. Assess participants’ experiences of the MBI to contribute toward further protocol refinements.

## Methods

### Participants

**Figure 1.**
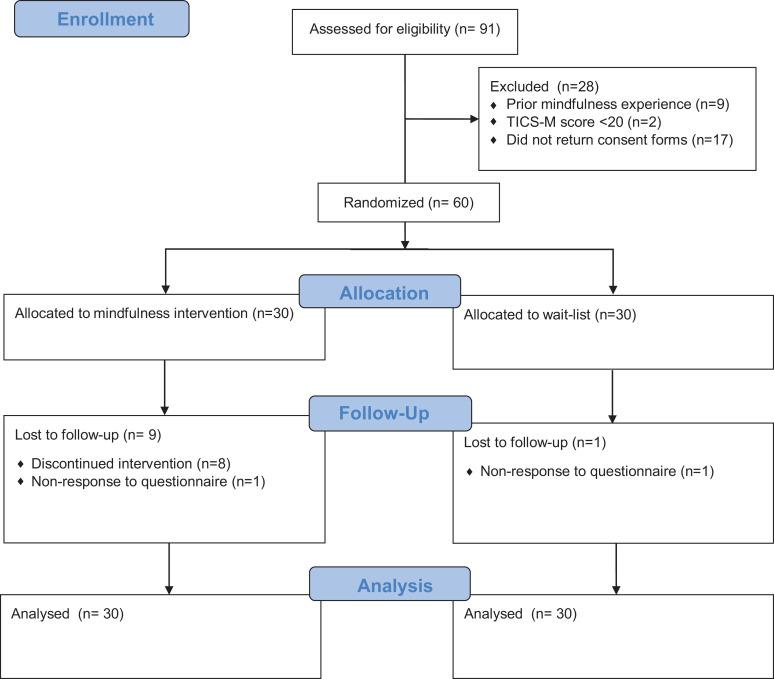
CONSORT flow diagram.

We employed a parallel-group, randomized control trial design. A trial of at least 30 participants is adequate for an efficacy trial.^26^ Enrolment of 60 participants was planned, anticipating a 10% dropout rate by 3 months. Participants were randomly assigned to either an 8-week MBI course (n = 30) or a wait-list control (n = 30). The control group was offered the opportunity to take part in the mindfulness courses at the end of the 3-month follow-up. CONSORT flow chart of the study is summarized in Figure 1.

Participants were recruited through adverts on the Parkinson’s UK and the Michael J. Fox Foundation websites and emails sent to the Parkinson’s UK Research Network. Recruitment took place between February and March 2016.

Participants included in this study: had a self-reported diagnosis of Parkinson’s by a neurologist or geriatrician, had a computer and internet access at home, they were able to communicate in English fluently and were stabilized on Parkinson’s medication, antidepressants or anxiolytics (if taken), indicated by a stable dose for a minimum of 1 month. Participants were excluded from the study if they self-reported a severe cognitive impairment that would make participation in the mindfulness sessions and home practice of mindful meditation problematic or distressing. Cognitive impairment was assessed using the Telephone Interview for Cognitive Status Instrument modified version.^27^ People with a score of less than 20 were excluded. People were also excluded if they reported any severe psychiatric conditions (e.g. psychosis, drug/alcohol abuse) that could potentially risk failure in the intervention or limit participation in the course; had severe hearing impairment, were participating in other psychological therapies at the time or had prior formal training in mindfulness methods or a current meditation practice.

### Standard Protocol Approvals, Registration, Funding and Patient Consents

Ethical approval was obtained from the City, University of London Psychology Ethics Committee (reference: PSYETH (S/F) 15/16 112) and registered with ClinicalTrials.gov (NCT02683330) in January 2016. Funding was obtained from Parkinson’s UK. The funders had no contribution to design, analysis or write-up of the study. More details on the protocol of the study have previously been published.^28^ All participants completed written informed consent.

### Randomization

Randomization took place once a cohort of 10 people had consented, were screened and had their baseline data collected. The trial coordinator (DS) conducted the randomization using a computer-generated randomly permuted blocks scheme. The randomization scheme was generated using the randomisation.com website. This randomization scheme allowed for keeping the groups in similar sizes, ensuring that the number of participants in each treatment group was roughly equal at any time during the trial. On receiving the randomization outcome from DS, the mindfulness facilitator (AB) notified participants of their group allocation, offered them a date for their first session and sent them web-cameras and headsets. Participants in the wait-list group were asked to refrain from any new mindfulness related activities during the trial. No participants of the control group reported mindfulness related activities during the waiting period. We used dummy codes in the data file, to ensure the researchers involved in data analysis were blinded to treatment group allocation until they completed the primary analyses. The nature of the intervention meant it was not feasible to keep the patients or treatment providers blind to treatment allocation.

### Mindfulness-Based Intervention

The MBI was delivered in 8 sessions over 8 weeks. The sessions lasted 1 hour and were held via videoconference through Skype, in groups of 5 people. The videoconferences were initiated by AB and were arranged at a day and time that was most suitable for all participants in the group. The facilitator of the group and the trial coordinator were available to help participants with setting up their equipment, use of Skype or other technical difficulties via telephone. Participants were given CDs with guided mindfulness meditations to follow. These guided meditations also addressed potential issues PwP may come across during practices, like resting tremor, spasms, fatigue and mind wandering.

The sessions were carried out based on a written manual — additional details on the content of the intervention can be found in the published protocol.^28^ The facilitator introduced the same topics at the same time-points to all groups. However, flexibility was allowed to repeat topics or discuss future topics earlier than expected, if needed. Participants were sent the mindfulness manual and a CD with the mindfulness meditations before their first session and were encouraged to read only the chapter relevant to the session each week. The manual contained 8 chapters. In each section, the homework for each week was outlined followed by a brief description of the week’s theme.

The intervention manual was based on the Mindfulness-Based Cognitive Therapy (MBCT) programme for depression developed by Segal, Williams and Teasdale.^15^ We tailored the programme to the needs of PwP. One of the main changes introduced was the reduction of time in mindfulness home practice to 20 minutes instead of the traditional 45 minutes. Recent evidence suggests that frequency and not the duration of the meditation practice is associated with positive psychological well-being.^29^ Further, we reduced the weekly group sessions from 2/2.5 hours to 1 hour. Each session contained all the elements of the sessions of the original protocol, all similar concepts were introduced, such as acceptance, relating to thoughts and self-compassion. However, the timings were significantly reduced. For example, all sessions started with a meditation practice that lasted 10 minutes instead of 45 minutes, followed by a 10 minutes inquiry compared to 20 minutes of inquiry in the original protocol. Then another short meditation practice followed this discussion, and in the end, homework for the next week was set. In previous studies the correlation between mean effect size and number of in-class hours was non-significant for both clinical and nonclinical samples, suggesting that adaptations which include less class time may be worthwhile for populations for whom longer time commitment may be a barrier to their ability to participate.^30^ Shortened class time has been used successfully, for example, in people with multiple sclerosis,^31^ and Parkinson’s.^16^ The manual also included Parkinson’s-specific examples. These changes made the intervention more accessible and relevant for PwP.

AB delivered the 8-week course. AB is a health psychologist who has completed teachers’ training to deliver mindfulness-based courses and has experience delivering mindfulness programs for people with neurological conditions via videoconference. LM, a clinical psychologist with experience in mindfulness programs for people with long-term conditions provided supervision.

AB recorded attendance and made notes on participation. In the case of non-attendance at sessions, AB contacted the participant to ascertain the problem and discuss a suitable solution, addressing any concerns. Because of the group setting of this intervention participants did not have the opportunity to reschedule a missed session.

## Wait-List/ Control

Participants allocated to the wait-list group (“WLC group,” hereafter) received the treatment they would normally expect within the NHS—typically a mix of clinical input and review from both primary and secondary care providers, according to individual health needs.

### Measures

Participants were assessed at 4-time points, baseline (pre-intervention), 4 weeks (mid-intervention), 8 weeks (post-intervention) and 20 weeks (follow-up). Assessments were completed online. The patient-reported outcome measures (PROMs) used for the assessment are detailed in the next sections.

#### Primary PROM

Hospital Anxiety and Depression Scale (HADS)^32^ was used to assess symptoms of depression (7 items) and anxiety (7 items). This scale is effective in assessing the severity of symptoms of anxiety and depression in both secondary and primary care patients,^33^ and the instrument has been proved to be reliable, valid, and responsive for use in PwP.^34^ Each item was scored on a scale of 0-3, with 3 indicating higher symptom frequencies. Scores for each subscale (anxiety and depression) were then totalled and ranged from 0-21.

#### Secondary PROMs

Brief Pain Inventory (BPI)^35^ measured pain severity/intensity, as well as pain interference with daily life across 7 domains. This is a numerical rating scale (scaled from 0 “does not interfere” to 10 “completely interferes”). This pain scale was used to assess the average pain associated with Parkinson’s.

Fatigue Severity Scale (FFS)^36^ has 9 items and was used to assess the impact of fatigue on the daily living of patients. It is comprised of 3 items related to physical impact, 3 items related to the psychological environment and 3 more generic items. Each item was rated on a 7-point Likert scale (1 = completely disagree—7 = completely agree). Mean scores of 4 or more defined significant fatigue.

Insomnia Severity Index (ISI)^37^ consists of 7 items that assess the nature, severity, and impact of insomnia in the last 2 weeks. Each item was rated on a 5-point Likert scale (0 = not at all—4 = extremely) with total scores ranging from 0 to 28, whereby higher scores indicated greater insomnia severity.

Parkinson’s Disease Activities of Daily Living Scale (PADLS)^38^ assessed difficulties in daily activities due to Parkinson’s. This 5-item scale was used to provide a single global rating of how patients perceive their illness, with higher mean scores indicating greater difficulty in activities of daily living. We used this measure as a proxy for disease severity.

#### HRQoL measures

Quality-Adjusted Life Years (QALYs) is the primary health outcome of interest in the cost-effectiveness analysis. One QALY is the equivalent to 1 year of life in full health. QALYs are therefore defined by weighting the life years by the HRQoL they will be lived in. HRQoL in this analysis is measured by the instrument EQ-5D-3 L.^39^ The instrument describes the quality of a health state by means of 5 dimensions (mobility, self-care, usual activities, pain/discomfort, and anxiety/depression). Each health profile described by means of the EQ-5D-3 L is mapped to a score (usually called “social tariff” or “utility index,” since the index is based on societal preferences over health states) that usually ranges from 0 to 1, with 0 indicating dead, and 1 representing full health. QALYs is a globally accepted measure of HRQoL in economic evaluations; in particular, the National Institute of Health and Care Excellence (NICE) in England evaluates the cost-effectiveness of health programs in terms of cost(£)-per-QALY, with an explicit threshold specified in the NICE assessment guidelines: an intervention that requires an investment in the range of £20,000 to £30,000 per QALY gained (or below) is likely to be funded by the NHS.^40^

### Feedback Interview Procedure

At the end of the 8-week intervention period participants in the mindfulness, group were also interviewed by an MSc health psychology student, who had no involvement in other aspects of the trial. Participants were asked to give open and honest accounts of their experiences and opinions of the intervention (see topic guide Table 1). All participants were asked the same questions using the same prompts. Questions addressed expectations regarding the intervention, experiences of the mindfulness sessions, features of the intervention liked and disliked, and the process of change (or not) as a result of the course. Each interview was digitally recorded. Interviews were anonymized and transcribed; any information that may have compromised confidentiality was omitted before the research team received the transcripts.

**Table 1.** Topic Guide for Participants’ Experiences With the Mindfulness Course.

Questions	Prompts
1. Can you start by telling me what you were expecting from the mindfulness sessions?	– What did you think the programme would be like? – In what ways (if any) did you think it might help you?
2. How did you find the programme overall?	– Tell me how you found your first session – Tell me about the other sessions – Tell me how you found the homework tasks
3. Can you tell me what you liked about the programme?	– What was helpful? Why? How? – Were there some sessions/ aspects more helpful than others?
4. Can you tell me what you disliked about the programme?	– What was unhelpful? Why? How? – Were there some sessions/ some aspects that were less helpful than others?
5. Tell me about anything that you feel has changed from having done the programme?	– Can you tell me what changed? (anything different in your day-to-day life, the way you are dealing with Parkinson’s?) – Can you tell me how you came to notice things changing? – Why/how do you think things changed?
6. Do you have anything else you would like to tell me about your experiences of this programme that haven’t already covered?	– What would you feed back to the people who put together the programme? – What advice would you give to people thinking about taking part in mindfulness-based programs?
7. What do you think of the questionnaires used and the overall set up of the study?	– How did you find participating in a course over Skype? – Any further comments regarding the questionnaires used?

### Data Analysis Plan

In order to test the feasibility of this trial, we examined the number of people expressing interest, screened and enrolled in the study during the recruitment period. We also looked at the percentage of people who were eligible of those screened and percentage of eligible participants who consented to take part in the study. We also looked at the number of participants who dropped out of the study for each group and the percentage of participants attending a different number of sessions. Statistical analysis was carried out in SPSS v24. All group comparisons were carried out on an intention-to-treat basis; that is, participants were analyzed in the group to which they were randomized. Intention to treat analyses are suggested to help overcome potentially biasing effects of noncompliance and missing data by including all participants. In order to include all participants, those with missing data had their missing values substituted using the Last Observation Carried Forward method. We have also conducted a sensitivity analysis, where unimputed data was analyzed. For missing items in each questionnaire, the missing items were replaced with the average score from the rest of the subscale or scale, as long as less than 5 scores were missing.

2 (group: Mindfulness, Wait-list) x 4 (time: baseline, mid-intervention, post-intervention, follow-up) mixed ANOVAs were conducted to see the effect of group allocation (between-subjects factor) and time (within-subjects factor) on both the primary and secondary outcome measures (distress, depression, anxiety, pain, fatigue, insomnia and impact of Parkinson’s on daily activities). Effect sizes (Cohen’s d and partial eta-squared) were also computed during analysis.

The EQ-5D-3 L questionnaire was collected at baseline, post-intervention (week 8), and the end of 3-month follow up (week 20). Each EQ-5D-3 L response was linked to a HRQoL weight, obtained from the UK social tariffs.^41^ We derived the average HRQoL reported by the MBI and WLC groups, at every data collection point. QALYs gained by the mindfulness intervention were computed as the differences in HRQoL changes between the MBI and the WLC groups, with respect to the pre-treatment HRQoL of each group. Our reference-case considered HRQoL gains during the treatment, and up to 12 weeks after the treatment. The estimate was obtained by adding the QALYs gained during the treatment (i.e. between-groups differences in the incremental HRQoL from baseline to week 8, multiplied by 56/365 years) and the QALYs gained from week 8 to week 20 (i.e. between-groups differences in the incremental HRQoL from week 8 to week 20, multiplied by 84/365 years).

The Incremental Cost-Effectiveness Ratio (ICER) was derived from data related to the costs of the programme and the estimates of “QALYs gained” described above. We performed univariate and probabilistic sensitivity analysis to explore the uncertainty around the ICER estimate. Detailed results are not presented here but they are available upon request.

#### Qualitative analysis plan

The interviews were analyzed using inductive thematic analysis. The inductive analysis is a process of coding the data without trying to fit it into a pre-existing coding frame, or the researcher’s analytic preconceptions.^42^ The analysis was conducted following Braun and Clarke’s guidelines.^43^ The analysis of the transcripts was conducted in parallel with ongoing data collection. First, each coding unit in the first transcript was given a code name, using vocabulary as close as possible to that used by participants themselves to avoid prematurely importing preconceptions into the analysis. This procedure was repeated on the second transcript. When the same themes recurred, they were provided with the same label. Initial codes were then applied systematically to the entire dataset, giving full and equal attention to each data item. As data analysis proceeded, codes were re-defined as new and alternative themes arose. Earlier transcripts were re-coded as codes were developed and refined. During this analysis, the validity of individual themes about the dataset was considered and also whether the themes reflected the data set as a whole. A detailed paper trail recorded the development of the codes and the relationship between the raw data and the refined themes and codes. Further, framework analysis techniques were used, where the final codes were tabulated to inspect the data for patterns and relationships in the themes between PwP.

## Results

Half of the sample were women (n = 30). The majority of the sample was married or co-habiting (n = 44, 73.3%), had college or higher education (n = 56, 93.3%) and were White British (n = 58, 96,7%). As shown in Table 2, the 2 groups were well matched, regarding gender, age, years since diagnosis, disease severity measured by PADLS and anxiety and depression measured by HADS, with no statistically significant differences between the groups. The sample size allowed us to include 6 intervention groups.

**Table 2.** Demographics and Baseline Clinical Characteristics of Participants.

Variable	Mindfulness (n = 30)	Wait-list (n = 30)	Statistical test
Gender, female (n, %)	13 (43.3)	17 (56.7)	^χ2^ = 1.067, p = .302
Age, in years (M, SD)	59.50 (11.12)	62.23 (8.96)	t(58) = 1.048, p = .299
Years since diagnosis (M, SD)	5.22 (3.55)	6.43 (3.85)	t(58) = 1.273, p = .208
Disease severity (PADLS) (M, SD)	2.10 (0.61)	2.13 (0.68)	t = .200, p = .0842
Anxiety (HADS) (M, SD)	8.70 (4.23)	7.73 (3.59)	t(58) = −.960, p = .341
Depression (HADS) (M, SD)	7.23 (3.46)	5.73 (3.00)	t(58) = −1.778, p = .08

### Trial Processes (Aim 1)

#### Recruitment, time and resources

One hundred five potential participants expressed interest in taking part in the study within 1 week of advertising. Ninety-one participants were screened for eligibility between February and August 2016. The intervention was delivered to groups between March and August 2016.

#### Appropriateness of eligibility criteria and refusal rates

Of the 11 people screened but excluded, 9 (81.81%) had previous experience in mindfulness training, and 2 (18.18%) had a low TICS score. Of the 80 eligible participants who received an invitation and a consent form to the study, 63 (78.75%) consented to take part in the study and 60 were randomized.

#### Retention rates

Eight participants dropped out of the intervention. Of these, 4 dropped out after attending 2 sessions, 2 participants dropped out of the intervention after attending one session, and 2 participants dropped out before attending any sessions. Three participants dropped out because they did not have time, one did not like mindfulness, one did not like using Skype, one became too ill to participate, one missed too many sessions due to other commitments, and one had a bereavement in the family.

All participants were encouraged to complete the follow-up questionnaires even if they had dropped out of the intervention. Four participants who dropped out completed the mid-intervention questionnaires, and one also completed the end of the intervention and follow-up questionnaires.

#### Adherence to mindfulness sessions

Twenty-two out of the 30 mindfulness participants (73.3%) attended 4 or more of the 8 sessions, 8 (26.67%) attended all sessions, and 5 (16.67%) attended 7 sessions, and 6 (20%) attended 6 of the 8 sessions. The main reasons people cited for not being able to attend were either problems with their computer or Skype (10 absences) or being away on holidays (10 absences). Other reasons included work commitments (6 absences), feeling ill (5 absences) or having a doctor’s appointment (5 absences), family commitments (4 absences) and forgetting about the session (2 absences).

The baseline level of disability did not predict the number of sessions attended (F(1,28) = 2.428, p = 0.13). Similarly, baseline anxiety (F(1,28) = 1.406, p = 0.246) and depression (F(1,28) = 0.286, p = 0.597) scores were not associated with the number of sessions attended.

### Potential Efficacy (Aim 2)

#### Descriptive statistics

As shown in Table 3, there were small differences in anxiety and depression scores for the mindfulness group compared with the control group. Additionally, there were small differences in pain, fatigue, insomnia and the impact of Parkinson’s between the 2 groups and corresponding small effect sizes (see Table 3). Results of the sensitivity analysis, where unimputed data was analyzed showed similar effect sizes and the results are presented in the online supplementary document.

**Table 3.** Estimated Post-Therapy Group Differences (Treatment Effects) for Primary, Secondary Outcomes.

		Mindfulness		WLC			
		Mean (SD)	CI (LB-UB)	Mean (SD)	CI (LB-UB)	Mean* diff	Effect size* Cohen’s d
Anxiety	baseline	8.70 (4.24)	7.26-10.13	7.73 (3.59)	6.30-9.17	−0.97	0.25
	mid-intervention	7.27 (4.88)	5.67-8.86	6.40 (3.80)	4.80-8.00	−0.87	0.20
	post-intervention	7.53 (4.22)	6.07-9.00	6.20 (3.75)	4.74-7.66	−1.33	0.33
	follow-up	6.97 (4.44)	5.44-8.49	6.17 (3.87)	4.64-7.69	−0.80	0.19
Depression	baseline	7.23 (3.46)	6.05-8.42	5.73 (3.00)	4.55-6.92	−1.50	0.46
	mid-intervention	6.27 (3.84)	5.02-7.51	5.13 (2.89)	3.89-6.37	−1.14	0.34
	post-intervention	5.53 (3.74)	4.26-6.80	5.33 (3.20)	4.06-6.60	−0.20	0.06
	follow-up	6.03 (4.13)	4.71-7.36	5.33 (3.06)	4.01-6.66	−0.70	0.19
Pain	Baseline	3.57 (2.01)	2.77-4.37	3.41 (2.38)	2.60-4.21	−0.16	0.07
	mid-intervention	3.34 (2.10)	2.53-4.16	3.56 (2.35)	2.74-4.37	0.22	0.10
	post-intervention	3.21 (2.22)	2.39-4.04	3.32 (2.31)	2.49-4.15	0.11	0.05
	follow-up	3.75 (2.17)	2.99-4.60	3.60 (2.47)	2.75-4.45	−0.15	0.06
Fatigue	Baseline	4.08 (1.30)	3.54-4.62	4.01 (1.62)	3.47-4.54	−0.07	0.05
	mid-intervention	4.18 (1.30)	3.63-4.73	3.69 (1.69)	3.14-4.24	−0.49	0.33
	post-intervention	4.20 (1.16)	3.68-4.71	3.96 (1.64)	3.44-4.48	−0.24	0.17
	follow-up	4.39 (1.30)	3.83-4.94	4.04 (1.72)	3.48-4.60	−0.35	0.23
Insomnia	Baseline	11.17 (5.38)	9.02-13.31	9.27 (6.31)	7.12-11.41	−1.90	0.32
	mid-intervention	16.10 (6.68)	13.65-18.55	15.20 (6.72)	12.75-17.65	−0.90	0.13
	post-intervention	16.00 (6.01)	13.73-18.27	15.40 (6.42)	13.13-17.67	−0.60	0.10
	follow-up	16.57 (6.01)	14.20-18.93	16.17 (6.89)	13.80-18.53	−0.13	0.06
Impact of Parkinson’s	Baseline	2.10 (0.61)	1.86-2.34	2.13 (0.68)	1.89-2.37	0.03	0.04
	Mid-intervention	2.07 (0.69)	1.82-2.32	2.03 (0.67)	1.79-2.30	−0.04	0.06
	post-intervention	2.00 (0.74)	1.73-2.27	2.10 (0.71)	1.83-2.37	0.10	0.14
	follow-up	2.03 (0.77)	1.73-2,34	2.23 (0.90)	1.93-2.54	0.20	0.24

* Mean difference and effect sizes reflect between groups differences.

#### Primary PROM

Table 4 presents mixed ANOVA results for depression and anxiety. When performing an intention to treat analyses, a significant main effect of time for both depression and anxiety scores was revealed, but no significant main effect of group and no significant interaction between group and time was found.

**Table 4.** ANOVA Results for Primary and Secondary Outcomes.

		*df*	*F*	*p*	*ηp^2^*
Anxiety					
	Time	3, 174	12.61	<.001	.1790
	Group	1, 58	.98	.325	.017
	Group x Time	3,174	.32	.809	.006
Depression					
	Time	2.611, 151.431	5.49	.002	.086
	Group	1, 58	1.16	.287	.020
	Group x Time	2.611, 151.431	2.08	.114	.035
Pain					
	Time	2.474, 143.493	2.23	.099	.037
	Group	1, 58	.00	.996	.000
	Group x Time	2.474, 143.493	.69	.533	.012
Fatigue					
	Time	3, 174	1.78	.154	.030
	Group	1, 58	.65	.424	.011
	Group x Time	3, 174	1.00	.392	.017
Insomnia					
	Time	3, 174	66.11	.001	.553
	Group	1, 58	.39	.533	.007
	Group x Time	3, 174	.89	.446	.015
Impact of Parkinson’s					
	Time	3, 174	.90	.446	.015
	Group	1, 58	.20	.659	.003
	Group x Time	3, 174	1.15	.330	.019

#### Secondary PROMs

Mixed ANOVA results concerning pain, fatigue severity, insomnia and the impact of Parkinson’s on daily living are also highlighted in Table 4. No main effect for time was revealed for pain, fatigue, and the impact of Parkinson’s. However, a significant main effect of time was present for insomnia scores. Despite this, there was no significant difference between group scores for any of the secondary measures and no significant interactions between group and time either.

### Potential Effectiveness and Cost-Effectiveness (Aim 3)

#### HRQoL measures

Both groups (MBI and WLC) experienced an increase in HRQoL post-intervention (week 8) and a decrease in the next 3 months (week 20). However, the MBI group did exhibited faster increase rate and slower decrease rate than the WLC group.

#### Costs

Since the MBI is complementary, we assumed that all the patients are following the usual care pathway. We made the conservative assumption that an MBI did not reduce the healthcare use of those receiving it. Therefore, the cost of receiving the MBI (a total of £240) was considered to be the only incremental cost between PwP in the WLC group and the MBI group.

#### Cost-effectiveness

**Figure 2.**
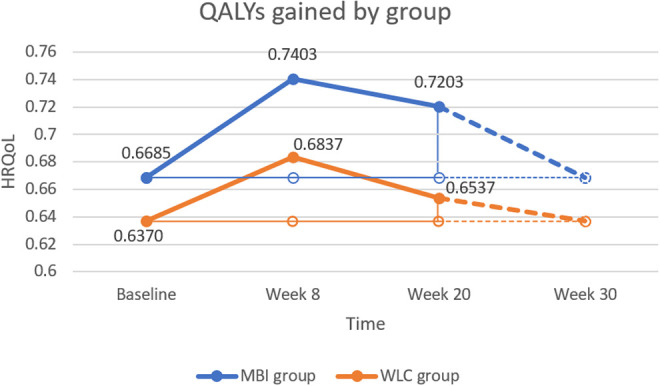
Change in health-related quality of life (HRQoL) in the mindfulness (MBI) and wait-list control (WLC) group.

The intervention provided a QALY gain of 0.0089 QALYs, which resulted from subtracting the 0.0109 QALYs gained by the WLC group from the 0.0197 QALYs gained by the MBI group, see Figure 2, where QALYs gained by group corresponds to the areas delimited by the solid lines in orange and blue. The incremental cost-effectiveness ratio was £27,107. This estimate was based on rather conservative assumptions: the MBI did not have any effect on HRQOL after week 20; changes in HRQoL were gradual (linear); the MBI did not reduce the healthcare use of those receiving it; and the HRQOL improvement in the 20 week period did not relate to the intervention. The HRQoL baseline was different for both groups; for this reason, the simple difference in QALYs between the groups (area between the blue and orange thick lines, in Figure 2) was not taken as a valid estimate.

### Participants’ Experiences (Aim 3)

All participants who completed the intervention were interviewed at the end of the mindfulness course (n = 25), plus one participant who dropped out of the study. People were asked about their experience participating in the online mindfulness training, what they found helpful and what not so helpful. Three main themes were identified from the interviews, the online nature of the intervention, group setting and mindfulness skills. Pseudonyms are used in the quotes provided to illustrate each theme.

#### Online nature of the intervention

Most participants reported some problems with Skype, with internet connection issues disrupting their sessions. Despite feeling that face-to-face sessions potentially could have been better, videoconferences were identified as convenient, especially for those with mobility restrictions.

As I say with my Skype connection, a pain in the bum, but certainly better than having to go backward and forward to a hospital for an appointment or anything like that (Kathryn, age 50).

The interviews also revealed that participating online helped form a unique bond since participants were more comfortable sharing intimate experiences remotely, from their own home.

So that sort of slightest distance with interaction, I found helpful. Because I think somehow when you are physically in the same space, you can more easily be intimidated by others; you can be more prone to groupthink, possibly. I think, as I say, that detachment of the Skype situation I thought worked quite well. (Rebecca, age 64)

#### Group setting

The participants enjoyed sharing their experience with the group and meeting others with Parkinson’s. They found talking about the exercises helped them express their feelings verbally and recognize their symptoms.

Yeah we discussed it at length and when we had a bit more of an explanation and when I heard colleagues in the class using different words to explain something it made sense to me. (Adam, age 52)

The sharing of experiences with others with the same condition led to a reduction of fear for future symptoms and disease progression, particularly for younger participants with fewer symptoms.

To get other people’s perspective because quite a few of them were members of Parkinson’s clubs whereas I’m not and erm I suppose at this stage I’m not…you know I’ve now looked at a group of people with Parkinson’s and do you know what I thought they’re not that bad are they. (Adam, age 52)

#### Mindfulness skills

Following the course, participants reported a decrease in anxiety and felt more in control of symptoms and aware of how the condition has made them feel. The mindfulness course also helped recently diagnosed people to accept their diagnosis.

By accepting it and letting it be as it is, you’re not worrying are you? (Josh, age 53)

I’ve always said, if that’s in describing our condition with other people who’ve got the same condition, we have to be transparent as well, and I think the mindfulness has enabled me to be more transparent with people about how my condition actually makes me feel a lot of the time. (Kathryn, age 50)

Overall, the experience of the intervention was reported as positive even by those who felt that they did not directly benefit from the practice of mindfulness.

we would say how you felt, what they did for you and when they didn’t do anything much for me, so I was quiet, I was struggling to say positive things, but I didn’t want to be totally negative, but I mean I would get something from them, just not as much as I would have liked. (Eleanor, age 54)

Participants found that the programme helped to develop a regular practice which led to improvements in their ability to focus. The mindfulness practice was found to have a positive effect on other activities such as exercise.

The yoga ones were fine because I do yoga, which helps. I think it helps me a lot, I do yoga once, maybe twice a week, and I get meditation at the end. Plus mindfulness comes in quite a lot to yoga in some of the exercises we do…I do benefit from going to yoga, and the mindfulness has come into it, and I am more aware of it now. So that is good. (Eleanor, age 54)

The body scan, in particular, was reported as a good tool to help relaxation, sleep and pain, especially for participants with less severe symptoms.

I thought that was practical because it’s literally for a few minutes…I mean that’s something I’d certainly take with me because it’s, it’s like err, like a brainstorm, like a personal brainstorming reset if you like. (Adam, age 52)

There were some barriers to formal practice relating to symptoms, but lack of time was the most significant barrier, especially when meditations were not benefiting the participant. Most participants had not developed a formal practice by the end of the programme but used the meditations for specific causes or symptoms.

It was the time it took to do it. And, and that, that remains, um, the time it takes to do it (Evelyn, age 68)

I found it difficult putting aside time, but I should have time, step back and do it…I was making myself do it…If I had seen it working I would have probably been more enthusiastic to do it. (Eleanor, age 54)

Participants approached the programme open-mindedly.

Maybe it’s because I had no expectations because I always try to, with my condition, I would try to have an open mind, and whenever I sort of looked into any sort of clinical trials, or any trials at all, I try to go in with a completely open mind, so that like I say, I don’t feel that my expectation gonna end up disappointed. (Kathryn, age 50)

However, those with anxiety found the programme more beneficial than those with other symptoms such as concentration issues.

well I know that my anxiety is Parkinson’s based. Um, so in some ways, that’s a sort of, um, that’s how I see the benefit of it. I see the benefit of the techniques. Because, I’ve been diagnosed 4 years, and my motor aspects are not well advanced. (Jim, age 67)

Some participants found the hour-long sessions and the programme overall too short for developing their mindfulness skills, reporting that it ended abruptly, leaving them without support.

I wonder actually if it might be useful to do the, like say the 8 weeks, and then, um, sort of wean, wean us off it rather than just suddenly stop…just have a how are you getting on, just a recap, and maybe just once actually. I don’t know, possibly in a way to give you something you’ve got to work toward (Elsa, age 61)

Participants suggested follow-up sessions may be beneficial. Alternatively, suggestions of where mindfulness is available to participants and reminders to keep in touch with other members of the group could be beneficial. They also reported that missing sessions, particularly the first session, negatively affected group bonding and progression within the programme.

it’s just that I thought that at the beginning it, it wasn’t so beneficial to miss it because, you know, if it had been later on in the course I would’ve caught up…it’s just that I might’ve met more people. I might’ve bonded better with the people in my group. (Evelyn, age 68)

## Discussion

To date, there is very little research in evaluating the effects of mindfulness in reducing anxiety and depression in PwP. The present study assessed the feasibility and potential efficacy of an online MBI specifically designed for people living with Parkinson’s. The study met the feasibility aims suggesting that the adapted online delivered MBI is acceptable to participants regarding willingness to be screened for and enter a randomized controlled trial, completing the intervention and follow up measures. Although the intervention did not show any significant effect in the selected PROMs, however the overall effect in HRQOL is shown to be positive. In addition, the qualitative study showed that mindfulness is a suitable and acceptable therapy for people living with Parkinson’s.

The current trial shows that the mindfulness courses were of great interest for people with Parkinson’s, as evidenced by the large number of potential participants that showed interest in taking part within a few days of the study being advertised and the willingness of people to be screened and wait for the mindfulness courses. Based on the qualitative findings, the participants enjoyed sharing their experience with the group and meeting others living with Parkinson’s. Participants also reported and that the intervention led to a reduction in fear of future symptoms and disease progression. These qualitative findings support previous research. For instance, several quantitative studies have found that MBIs help symptom management and control,^44^ as was identified in the present study’s interview. The present study thus helps to build on existing literature suggesting that MBIs are well accepted by PwP and can have some positive effect. As shown in the qualitative interviews, the fact that the courses were tailored for PwP made those courses particularly appealing. The mindfulness course offered in the current trial was customized to address specific issues that PwP face.

Further, having group discussions with other PwP appeared to have the additional advantage of reducing the sense of isolation and reinforcing a sense of camaraderie with people faced with similar challenges. People felt less alone and were able to explore further how mindfulness could be used to address some of their challenges. These group effects may have enhanced the effectiveness of the MBI. PwP valued the group dynamic and found it one of the most helpful elements of the programme. Qualitative research of mindfulness programs illustrates the benefits of the group environment in offering a sense of community and support,^21,45,46^ opportunities for learning from others,^47-49^ and motivation to maintain mindfulness practise.^45,48,50^ There is less clarity about the potential benefits and disadvantages of homogeneous groups.^51^ Our qualitative findings provide support for the positive effects of a homogeneous mindfulness group. We did not assess the group effects quantitatively. To the best of our knowledge, there is only one large quantitative study (n = 606 from 59 groups) that has looked at group processes and found a significant correlation between group-level variance and improved outcomes in participants’ levels of psychological distress.^52^ Future trials should measure social support as a direct or indirect effect of mindfulness courses. The role of group processes should also be explored by introducing a non-mindfulness-based control group.

This study showed that an online MBI is feasible for PwP. A significant potential barrier to such intervention within a population bound by mobility restrictions is the weekly frequency of meetings for 8 weeks. A mindfulness course delivered via videoconferencing removes logistical barriers that a face to face intervention can pose and can make it easier for people to sign up for such interventions and attend sessions. Further, of the 240 sessions (30 participants x 8 sessions each participant) only 10 (4%) were missed due to technical difficulties, showing that the technology used was not a major barrier for people to participate in the MBI. In the qualitative interviews, people were probed to discuss difficulties with Skype or the technology used. Even though most participants mention glitches during the sessions and some confusion over how to set up Skype and how to join the group, they also talked about how they would not have the chance to participate in such a course at all if it was delivered face to face. Videoconference applications are becoming more common, and at the moment there is a large number of online applications that offer videoconferencing; Facebook messenger, FaceTime, WhatsApp, Viber, Zoom, Google hangouts to name a few. These services are becoming easier to use, more sophisticated, with less technical glitches.

A potent ingredient for any mindfulness course is the daily meditation practice. A meta-analysis of 43 studies (n = 1427) of MBIs^53^ showed that participants’ average mindfulness home practice time equated to 30 min per day, 6 days per week and the extend of practice positively correlated with intervention outcomes. The small body of studies with reduced home practice identified by the meta-analysis^53^ showed that participants in these studies practiced significantly less overall than those asked to practice for the standard amount of time (i.e., 151 min vs 174 mins per week). In the current intervention, participants were asked to complete weekly homework practices, related to the week’s session, and record them in a diary. However, many diaries were incomplete, and some entries appeared to have been created at the last minute. This made it difficult to assess whether homework had been completed, and it might be an indication that participants did not engage with the homework practice. Further, participants reported during the qualitative interviews that they had difficulties keeping up with the daily mindfulness practice. Future research studies need to identify ways to facilitate daily meditation practice and include elements that can ensure that daily meditation becomes a habit, an automated response to a predetermined cue.

This efficacy trial also showed issues that need to be addressed in a future larger trial. Most participants who dropped out did so after attending 0-2 sessions. A taster day to show potential participants what an MBI involves could have increased the attendance rates and reduced the dropout rates. The taster day could be a short session in a group setting where the facilitator explains in more detail what mindfulness is and the type of content and format of each session, followed by a short mindfulness practice and an inquiry after the practice. The taster session may end answering questions of the potential participants and addressing some frequently asked questions misconceptions, like the origins of mindfulness, whether mindfulness is passive resignation, whether mindfulness is a relaxation technique or whether participants will be able to “control” their body if they learn to “control” their mind. This taster session will give participants a better idea of what mindfulness is and may help them decide whether they would like to commit to the mindfulness course or not.

The present study did not find any significant effects of mindfulness on depression and anxiety. However, the descriptive statistics showed that both groups had overall low scores of anxiety and depression, thus leaving little scope for significant improvement. After consultations with PwP, it was decided not to have depression and anxiety symptoms as inclusion criteria.^18^ To retain the inclusivity of these intervention but also be able to quantify their effectiveness, future studies might need to consider alternative ways to measure outcomes, for example a goal oriented outcome measure, where each participant sets their personal goals at the beginning of the intervention and participants are encouraged to use the mindfulness techniques to meet their personal goals. Other widely used measures of disability and change that can be used as outcome measures in future psychological interventions in Parkinson’s may also include the World Health Organization Disability Assessment Schedule (WHODAS),^54^ the Patient-specific functional scale,^55^ the Warwick-Edinburgh Mental Well-Being Scale^56^ and the Patient Activation Measure.^57^ Also, trials could include explicitly planned moderator analyses, using baseline anxiety and depression as predictors of differential effects. The participants found the programme as well as some of the sessions too short. Modifying the sessions by reducing their length might not have been appropriate for this population. The participants may not have had time to explore and process the concepts introduced in the sessions. It could be argued that the Parkinson’s population may need as much, if not more, a mindfulness practice time to learn and practice mindfulness of that of the general population, to benefit. Therefore, we need to look at new ways to overcome difficulties the Parkinson’s population may face with practice, such as concentration and fatigue, for example, having more extended sessions with more breaks within them or more frequent but shorter sessions, or provide other supplemental or optional additional mindfulness training material that those who want or need it could access, including pre-recorded audio or video-based exercises. These changes would allow more considerably more time for sharing experiences while accommodating the needs of PwP.

Moreover, we have generally observed an increase in HRQoL for the WLC group from baseline to post-intervention. Even though we cannot prove the causality, it is plausible that the HRQoL gain was linked to the intervention. Although the WLC group did not have the chance to practice mindfulness systematically, it has been found that participants in control groups show some improvement in psychological outcomes and these effects are larger when participants are allocated in waiting-list groups.^58^ It seems that the hope of getting the intervention in the end of the trial can have positive psychological effect. Thus, being involved in the trial per se is likely to lead to a positive impact. Further, our reference case estimated a £27,107 cost-per-QALY of the intervention under the most conservative assumption (WLC group increasing self-reported HRQoL for reasons other than the intervention). If we assumed that at least a 15% of the increase in HRQoL for the WLC group is linked to the intervention, the cost per additional QALY gained from the intervention would drop below the £20,000. Our reference case £27,107 cost-per-QALY is based on the conservative assumption that there is no effect after week 20. If we assume that the intervention still has a positive effect in the HRQoL of participants (in both groups) for 10 additional weeks (in the way described by dashed lines in Figure 2), then the cost-per-QALY would also drop below £20,000. Therefore, the reference case estimate could be taken as an upper boundary, since MBI is likely to be more cost-effective than the estimates reported here.

Additionally, future research could also attempt to trial the recommendations to the MBI, revealed in participants’ interviews. A follow-up session after the course is completed may help to encourage the sustained practice of mindfulness, leading to a more meaningful and longer-lasting change in PwP. A decrease in HRQoL occurs immediately after the treatment, and there was no recap session during the 3-month follow up. Therefore, it is reasonable to assume that having recap sessions during the 3-month follow up period will slow down the decrease of HRQoL, or in a stronger assumption, the time-to-time recap will enable the patients to maintain the highest level HRQoL. Follow-up sessions could be built into the intervention by allocating a lead participant in the group and encouraging the group to continue meeting on a regular basis.

Several additional limitations in this study should be acknowledged. Due to the nature of recruitment, diagnoses were self-reported and not verified. The sample was a selective one, including people interested in mindfulness and with the devices and skills to use Skype. Naturally, this was a feasibility study and small in size, so the reliability, particularly for the treatment effects, will need to be investigated further in future more extensive studies. This study represents only a limited test of potential efficacy and generalizability may be limited.

## Conclusions

The present study found evidence that an online MBI is feasible and could work for a larger-scale trial. Preliminary estimations suggest that the intervention is likely to be cost-effective, compared with a waiting-list group that received only usual care. Further, including participants with elevated levels of anxiety and depression might increase the apparent efficacy of the intervention. Follow-up sessions could have supported future mindfulness practice. These adaptations may help future MBIs achieve more significant benefit for PwP.

## Supplemental Material

Supplemental Material, sj-pdf-1-jgp-10.1177_0891988720988901 - Acceptability and Feasibility of a Mindfulness Intervention Delivered via Videoconferencing for People With Parkinson’s Supplemental Material, sj-pdf-1-jgp-10.1177_0891988720988901 for Acceptability and Feasibility of a Mindfulness Intervention Delivered via Videoconferencing for People With Parkinson’s by Angeliki Bogosian, Catherine S. Hurt, John V. Hindle, Lance M. McCracken, Debora A. Vasconcelos e Sa, Sandra Axell, Katy Tapper, Jemima Stevens, P. Shashi Hirani, Marya Salhab, Wenrong Ye and Patricia Cubi-Molla in Journal of Geriatric Psychiatry and Neurology
