# Supplementary material for: Acceptability and Feasibility of a Mindfulness Intervention Delivered via Videoconferencing for People With Parkinson’s
Source: J Geriatr Psychiatry Neurol. 2021 Jan 28;35(1):155–67. doi: 10.1177/0891988720988901 (PMC8678660; doi:10.1177/0891988720988901)
Supplement: Supplemental Material, sj-pdf-1-jgp-10.1177_0891988720988901 - Acceptability and Feasibility of a Mindfulness Intervention Delivered via Videoconferencing for People With Parkinson’s [file sj-pdf-1-jgp-10.1177_0891988720988901.pdf]

Supplementary table of unimputed data (sensitivity analysis). Estimated post-therapy group differences (treatment effects) for primary, secondary outcomes

		Mindfulness (n=22)		WLC (n=27)			
		Mean (SD)	CI LB-UB	Mean (SD)	CI LB-UB	Mean* diff	Effect size* Cohen's d
Anxiety	baseline	8.18 (3.86)	6.54-9.82	7.77 (3.76)	6.30-9.25	0.41	0.11
	mid-intervention	6.22 (4.37)	4.44-8.01	6.29 (3.98)	4.68-7.91	-0.07	0.02
	post-intervention	6.77 (3.49)	5.17-8.37	6.11 (3.92)	4.66-7.55	0.66	0.18
	follow-up	6.00 (3.66)	4.36-7.64	6.29 (3.94)	4.82-7.77	-0.29	0.08
Depression	baseline	7.09 (3.11)	5.74-8.44	5.67 (3.16)	4.45-6.88	1.42	0.45
	mid-intervention	5.95 (2.98)	4.67-7.24	5.00 (3.01)	3.89-6.16	0.95	0.32
	post-intervention	5.00 (2.63)	3.70-6.30	5.15 (3.31)	3.97-6.32	0.53	0.05
	follow-up	5.68 (3.44)	4.26-7.10	5.29 (3.20)	4.01-6.58	0.39	0.12
Pain	Baseline	3.44 (1.90)	2.51-4.37	3.21 (2.35)	2.37-4.05	0.23	0.11
	mid-intervention	3.21 (2.02)	2.25-4.15	3.37 (2.34)	2.52-4.23	-0.16	0.07
	post-intervention	2.93 (2.22)	2.02-3.85	3.04 (2.13)	2.22-3.86	-0.11	0.05
	follow-up	3.66 (2.11)	2.68-4.64	3.40 (2.42)	2.52-4.29	0.26	0.11
Fatigue	Baseline	3.89 (1.24)	3.28-4.51	3.95 (1.55)	3.95-4.49	-0.06	0.04
	mid-intervention	3.98 (1.36)	3.33-4.63	3.57 (1.63)	2.98-4.16	0.41	0.27
	post-intervention	4.03 (1.17)	3.42-4.64	3.87 (1.59)	3.33-4.43	0.16	0.11
	follow-up	4.28 (1.38)	3.62-4.95	3.99 (1.67)	3.39-4.59	0.29	0.19
Insomnia	Baseline	11.04 (5.05)	8.49-13.60	9.48 (6.58)	7.18-11.78	1.56	0.26
	mid-intervention	16.45 (6.32)	13.68-19.23	16.00 (6.57)	13.50-18.50	0.45	0.07
	post-intervention	16.50 (5.27)	13.92-19.08	15.85 (6.56)	13.52-18.18	0.65	0.11
	follow-up	17.27 (5.17)	14.62-19.92	16.85 (6.89)	14.46-19.24	0.42	0.07
Impact of Parkinson's	Baseline	2.00 (0.55)	1.86-2.34	2.15 (0.66)	1.89-2.37	-0.15	0.25
	Mid-intervention	1.95 (0.67)	1.82-2.32	2.04 (0.65)	1.79-2.30	-0.09	0.14
	post-intervention	1.86 (0.73)	1.73-2.27	2.11 (0.70)	1.83-2.37	-0.25	0.35
	follow-up	1.95 (0.74)	1.73-2,34	2.26 (0.90)	1.93-2.54	-0.31	0.38

**Mean difference and effect sizes reflect between groups differences*
